# Gas chromatography-mass spectrometry profiling and analgesic, anti-inflammatory, antipyretic, and antihyperglycemic potentials of* Persea americana *

**DOI:** 10.22038/ijbms.2021.53390.12016

**Published:** 2021-05

**Authors:** Amany Hamouda Mahmoud, Mamdouh Nabil Samy, Amira Samir Wanas, Mohamed Salah Kamel

**Affiliations:** 1 Department of Pharmacognosy, Faculty of Pharmacy, Minia University, Minia 61519, Egypt

**Keywords:** Analgesic, Antihyperglycemic, Anti-inflammatory, Antipyretic, GC-MS analysis, Persea americana

## Abstract

**Objective(s):**

The present study determines the chemical constituents of *Persea americana *using gas chromatography-mass spectrometry (GC-MS) and its different activities.

**Materials and Methods:**

Air-dried powdered leaves of *Persea americana* were extracted by 95% methanol and fractionated consecutively with petroleum ether, chloroform, and ethyl acetate. The saponifiable matter, EtOAc and aqueous fractions were subjected to GC-MS. The analgesic, anti-inflammatory, antipyretic, and antihyperglycemic properties of extracts, different fractions, and crude polysaccharide were determined by hot plate, carrageenan-induced paw edema, yeast-induced pyrexia, and alloxan-induced hyperglycemia methods, respectively.

**Results:**

Fourteen fatty acid methyl esters were identified in GC-MS-based profiling of the saponifiable matter. Alongside, 13 compounds were determined from EtOAc fraction and 6 compounds from the aqueous fraction of *P. americana *leaves. The ethyl acetate fraction and total stem extract displayed high anti-inflammatory potential with percentage of paw edema reduction by 48.99 and 47.54 %, respectively, compared with that of indomethacin (42.90 %). The ethyl acetate fraction and total stem extract revealed the highest analgesic activity with 137.95 and 137.12 % percent of protection against external stimulus, respectively. Investigation of antipyretic efficiency showed the total stem extract and crude polysaccharides attained normal temperature after 3 hr, which was very close to that of acetylsalicylic acid. The total leaf and stem extracts displayed significant antihyperglycemic activity with significant reduction in the level of blood glucose by 76.67 and 59.05 %, respectively.

**Conclusion:**

*P. americana *had analgesic, anti-inflammatory, antipyretic, and antihyperglycemic properties, which refer to its bioactive metabolites.

## Introduction

The Lauraceae, also known as the Laurel family, is an extensively distributed family that comprises the true laurel and the closest relatives. It consists of more than 3000 species of flowering plants, distributed in more than 50 genera. The species grow mostly in tropical and warm temperate areas, particularly in South America and Southeast Asia (1). 

*Persea *genus is composed of around 150 species of evergreen trees. *P. americana *Mill (synonym: *Persea gratissima*), the avocado, which is a perennial tree endogenous to Central America and Mexico and spread throughout the world in subtropical and tropical regions, is the best-known member of the genus. Avocados are commercially important and are cultivated across the world in tropical and Mediterranean climates. The avocado fruit is a large, single-seed edible drupe often referred to as an avocado pear or alligator pear (owing to the green rough skin and shape of some cultivars) (2, 3). 

The trees of avocado are susceptible to nutritional, fungal, bacterial, and viral diseases. All plant parts can be affected by the disease, causing rotting, cankers, spotting, pitting, and discoloration (4).

In the traditional medicine of Africa and Latin America, it is used for the treatment of hypertension (5), renal disorders (6), and diabetes (7, 8), and for analgesic and antipyretic objectives (9). The leaves were chewed by humans as a cure for pyorrhea, and their aqueous extract displays extended hypotensive potential. They exhibited vasorelaxant, anti-diabetic, anti-convulsant, and anti-inflammatory properties (10). 

The skin, bark, leaves, and pit of avocado are reported to be toxic to animals: cattle, goats, rabbits, cats, and dogs (11), which may be seriously injured or even killed when they eat them. Avocado fruit is toxic to certain birds and is classified as poisonous to many animals including horses, cats, and dogs by the American Society for the Prevention of Cruelty to Animals. The leaves of avocado have a toxic fatty acid derivative called persin, which in adequate quantity can induce gripes in horses with no veterinary therapy and lastly death (12). 

Phytochemical investigation of Persea Americana leaves revealed the presence of several constituents including triterpene glycosides, coumarins (13), saponins, alkaloids, tannins (13, 14), reducing sugars (14), and flavonoids (15, 16).

Therefore, this study aims to determine the analgesic, anti-inflammatory, antipyretic and antihyperglycemic activities of total methanolic extract, its different fractions and crude polysaccharides, in addition to characterization of fatty acids in petroleum ether fraction and chemical constituents of EtOAc and aqueous fraction by GC-MS analysis.

## Materials and Methods

***Plant material***

The leaves and stems of *P. americana *were gathered from El-Zohria botanical garden, Giza, Egypt. It was cordially identified by Dr Nasser Barakat, Professor of Botany, Faculty of Science, Minia University. A voucher sample (Mn-ph-Cog-012) was saved in Pharmacognosy Herbarium Department, Faculty of Pharmacy, Minia University, Minia, Egypt.

***Preparation of plant extracts and different fractions***

The air-dried powdered *P. americana *leaves (2.5 Kg) were macerated with 95% methanol and concentrated to give 320 g of methanolic extract under reduced pressure, which was postponed in the smallest quantity of water and partitioned consecutively with petroleum ether, chloroform, and ethyl acetate. The soluble fractions were concentrated to produce petroleum ether (104 g), chloroform (8 g), and ethyl acetate fractions (34 g) under reduced pressure. To obtain the aqueous fraction, the remaining mother liquor was concentrated. 

The air-dried powdered stems (250 g) of *P. americana *were macerated with 95% methanol and concentrated under reduced pressure, yielding methanolic stem extract (40 g). 

***Preparation of the crude polysaccharides of the stem***

 The air-dried powdered stems of *P. americana* (250 g) were macerated for 24 hr in 500 ml distilled water, afterward filtered and concentrated to 100 ml. The aqueous extract was added stepwise to 1500 ml of MeOH and shaken vigorously, allowed to settle then filtered through sintered glass under vacuum. The obtained residue (17 g) was dried in the vacuum oven and left for further investigation (17). 

***Preparation of the fatty acids***

About 2 g of the dried petroleum ether extract of *P. americana* leaves was refluxed in a boiling water bath with 50 ml of N/2 alcoholic potassium hydroxide for around 8 hr for saponification. Most of the alcohol found was distilled off and the solvent remained was diluted with double its volume of water and extracted many times with chloroform until exhaustion. 

The alkaline aqueous solution (soap) was acidified with sulfuric acid (10%). The released fatty acids were extracted with consecutive minor volumes of chloroform. The mixed extracts of chloroform were washed with distilled water until the washings became neutral to litmus paper. The chloroform was evaporated and the residue of the total fatty acids was dried by calcium chloride, producing semisolid brown color (18). 

*Preparation of fatty acid methyl esters*

The fatty acids were transformed into their methyl esters via refluxing with 50 ml of absolute methanol and 1.5 ml concentrated sulfuric acid for 2 hr in boiling water bath. Most of the alcohol was evaporated, and the residue was diluted using distilled water and extracted with many portions of chloroform. The mixed chloroform fractions were washed with distilled water until the washing was free from acidity. The chloroform was evaporated and the residue was dried overnight over calcium chloride and then retained for further examination (18).

***GC-MS analysis of fatty acid methyl esters***

Shimadzu GC-MS with headspace system (Japan) was used. The Column of Rtx-Ms 30-meter length, 0.25 mm in diameter, and 0.25 um film thickness were used. The temperature of the column was programmed to begin from 70 °C and gradually increased to 220 °C throughout the experiment time (26 min). The detector temperature was 240 °C and the sample was injected as 1 µl at 240 °C. The total ion chromatograms and mass spectra were documented in the EI (Electron Impact) mode of ionization with a mass range of 50–500 m/z.

***GC-MS analysis of ethyl acetate and aqueous fractions***

The GC-MS was a Perkin Elmer Clarus 500 (Perkin Elmer, Shelton, CT, USA). Turbo Mass version 5.4.2.1617 was used as software controller/integrator. The GC capillary column used was an Elite-1 column, Crossbond® 100% dimethylpolysiloxane (30-meter × 0.25 mmID × 0.25 μmdf, Perkin Elmer). Helium (purity 99.9999%) was the carrier gas with a flow rate of 0.9 ml/min. The temperature of the source was 270 °C. The temperature of the GC line was 210 °C. There was 70 eV of electron energy, and 100 v of trap-emission. The oven was programmed to be the initial temperature ranging from 70 °C (held 2.0 min) to 150 °C (rate 10 °C/min, held 5.0 min), accompanied by a rise to 220 °C (rate 10.0 °C/min, held 5 min), after that increased to 280 °C (rate 20 °C/min, held 2.0 min). The temperature of the injector was 270 °C. The volume of injection was 1.0 µl, with a split ratio of 40:1. The total ion chromatogram (TIC) was applied to obtain the samples. MS can be from 40 to 350 m/z (500 scan/sec). Turbo Mass software was used to identify the closed peaks acquired from the MS chromatogram of the analyzed samples, after an average TIC scan of each peak, at specific retention times was saved.

***Animals***

The animals utilized in the present experiment were adult male albino rats weighing 120–170 g (for anti-inflammatory activity), 150–200 g (for anti-diabetic activity), and 200–250 g (for antipyretic and analgesic activities). They were housed and bred under uniform environmental conditions, fed standard diets, and acclimatized to the environment for at least one week before the experiments were conducted. The animals were only handled at the time of experiments and during cage cleaning. All conditions were made to minimize animal suffering. The research was carried out following the Committee of Institutional Animal Ethical, Department of Pharmacology and Toxicology, Faculty of Pharmacy, Minia University, Minia, Egypt.

***Anti-inflammatory activity (carrageenan-induced rat paw edema test)***

The anti-inflammatory efficiency was determined by paw edema induced by carrageenan. Male albino rats (120–170 g, each), were randomly distributed into 8 groups (5 animals per group). The different extracts and fractions were diluted in CMC (0.5% carboxymethylcellulose solution in distilled water) and administrated orally to rats. Rats received, after 1 hr, injection of 0.1 ml of carrageenan suspension (1% w/v in normal saline solution) into the subplantar area of the right hind paw. The control group was injected with 0.1 ml normal saline solution and given orally 10 ml/kg of the vehicle (CMC). While the standard group was given indomethacin at a dose of 8 mg/kg, p.o., and the test groups received 100 mg/kg of the different extracts and fractions.

Edema measurements were determined in mm with a plethysmometer at 0, 0.5, 1, 2, 3, 4, and 5 hr after carrageenan injection. The anti-inflammatory activity of the tested extracts and fractions was estimated by comparing the magnitude of paw swelling in the pretreated animals and that induced in control groups (19).

***Analgesic activity (hot-plate method)***

The hot-plate test method was employed in this study to evaluate the analgesic activity of different fractions of *P. americana*.

The test was performed on rats (200–250 g, each). The paws of rats were very sensitive to heat at temperatures that didn’t damage the skin. The responses were jumping, withdrawal, and licking of the paws. The hot plate, which was commercially available, consisted of an electrically heated copper plate. The temperature was controlled at 55 °C.

The animals were grouped into eight groups (five animals each). The control group was given 10 ml/kg of 0.5% CMC orally, while the standard group was given acetylsalicylic acid (100 mg/kg, p.o). The test groups were administered the different extracts and fractions at doses of 100 mg/kg. Each animal was placed on the hot plate in order to obtain the animal’s response to heat-induced pain. Jumping was taken as an indicator of the animal’s response to heat-induced pain. The individual rats were placed on the hot plate and were immediately removed as soon as responses like jumping or licking of the paws were observed. A cut-off time of 10 sec was followed to prevent any thermal damage to the paws. The time taken for each rat to jump out (reaction time) was noted and recorded by stopwatch in seconds at 0.5, 1, 2, 3, 4, and 5 hr following the drug. After each test, the hot-plate was cleaned with wet paper towels (20).

***Antipyretic activity (yeast-induced pyrexia)***

For the antipyretic study, the procedure of yeast-induced fever was adopted. The test was performed on rats (200–250 g, each) by subcutaneous injection (in the back, below the nape of the neck) of 20% aqueous suspension of yeast in a dose of 10 ml/kg to induce pyrexia. 

The animals were grouped into eight groups (five animals each). The control group was given 10 ml/kg of 5% CMC orally, while the positive control group was given acetylsalicylic acid (100 mg/kg, p.o). The test groups were administered the different extracts and fractions at doses of 100 mg/kg. The rectal temperature of each animal was recorded by inserting a thermometer 2 cm into the rectum at 0.5, 1, 2, 3, 4, and 5 hr after administration of the tested extract, fractions, and the reference drug (21).

***Antihyperglycemic activity (Alloxan-induced hyperglycemia)***

 The male rats (150–200 g, each) were allowed to fast for 24 hr prior to the experiment, and then a single dose of intraperitoneal injection of alloxan (120 mg/kg body weight) was introduced. After 72 hr of alloxan injection, hyperglycemia was confirmed by testing blood sugar level, using an Accu-chek active test strip in Accu-check active test meter to monitor the blood sample from the tail vein. Rats whose glucose level was above 180 mg/dl were considered diabetic. The control group was given 10 ml/kg of the vehicle (0.5 % CMC), while the standard group was treated with glibenclamide (0.5 mg/kg body weight orally). The test groups were administered the different extracts and fractions at dose of 100 mg/kg (19).

***Statistical analysis***

The findings of all biological experiments were expressed as means±SEM. In contrast to the control group, one-way analysis of variance (ANOVA) accompanied by Dunnett’s test was used to assess significance. *P-*values of less than 0.05, 0.01, and 0.001 were considered to be significant (*P<0.05, **P<0.01, and *** *P*<0.001). For statistical calculations, Graph Pad Prism 5 was used (Graph pad Software, San Diego California, USA).

## Results

***GC-MS analysis of fatty acid methyl esters***

GC-MS analysis of the saponifiable matter of leaves of *P.* *americana* (Table 1), exposed the presence of 14 compounds, of which 6 were recognized as saturated long-chain fatty acids that constitute about 67.6 % of the total fatty acids. On the other hand, 32.4 % of the total fatty acids were determined as unsaturated long-chain fatty acids. The identified saturated fatty acid methyl esters including hexadecanoic acid methyl ester, octadecanoic acid methyl ester, tetradecanoic acid methyl ester, and heptadecanoic acid methyl ester which were the major compounds (55 .07%, 4.89%, 4.30%, and 1.86, respectively). Furthermore, 8,11,14-docosatrienoic acid methyl ester, 9,12-octadecadienoic acid methyl ester, 4,8,12-trimethyltridecan-4-olide, and 9-octadecenoic acid methyl ester are the major identified unsaturated fatty acid methyl esters (14.89%, 9.92%, 3.39%, and 1.74%, respectively), while the other characterized fatty acid methyl esters were in trace amounts.

***GC-MS analysis of ethyl acetate and aqueous fractions***

The ethyl acetate and aqueous fractions of leaves of *P. americana* were analyzed by GC-MS analysis using conditions given below. The different fractions have been subjected to silylation by N-methyl-N-trimethylsilyl-trifluoroacetamide (MSTFA) before carrying out GLC analysis. The identification of the compounds was done by direct comparison of their retention times and fragmentation pattern of every component with those of reference ones. It is clear that the identified compounds from ethyl acetate and aqueous fractions are different except for glycerol that was detected in both of them.

Thirteen compounds have been determined from EtOAc fraction (Table 2), including four organic acids (Benzoic acid, 4- amino-butanoic acid, 3-deoxy-D-arabino-hexonic acid-γ-lactone, and butanedioic acid), two volatile compounds (caryophyllene oxide and borneol), and three carbohydrates (1-methyl-hexafuranoside, D-xylopyranose, and lyxose), in addition to three alcohols (3-Desoxy pentitol, phytol, and glycerol).

From the aqueous fraction, 6 compounds have been determined (Table 3), of which two organic acids (malonic acid and succinic acid), two alcohols (glycerol and ribitol), in addition to N-trifluoroacetyl D- glucosamine, and 1, 2-dihydroxy-2-phenyl propane.

***Anti-inflammatory activity***

Different extracts and fractions of leaves and stems of *P. americana* exhibited anti-inflammatory property by inhibition of carrageenan-induced edema (Tables 4 and 6).

After 3 hr, the anti-inflammatory efficiency of total stem extract and ethyl acetate fraction of leaves (41.45 and 25.78 %, respectively) was almost higher than that of indomethacin (21.08 %), while crude polysaccharides of the stem showed inhibition of paw edema similar to that of indomethacin (21.08 %). On the other hand, the other fractions didn’t show high activity. 

The inhibition of paw edema by ethyl acetate fraction of leaves and total stem extract was increased to 48.99 and 47.54 %, respectively, after 5 hr, compared with that of indomethacin (42.90 %), revealing their long duration of action. In addition, petroleum ether fraction of leaves and crude polysaccharides showed significant anti-inflammatory effects with a percentage of inhibition of 38.55 and 30.29 %, respectively, after 5 hr.

***Analgesic activity (hot-plate method)***

The different extracts and fractions of *P. americana* leaves and stems exhibited marked and significant analgesic activity (Tables 6 and 7).

After1 hr, the ethyl acetate fraction of leaves exhibited significant analgesic activity (17.63 sec) that was nearly close to acetylsalicylic acid activity (17.16 sec), while crude polysaccharides of the stem, total stem extract, aqueous and petroleum ether fractions of leaves showed analgesic activities of 15.73, 14.10, 13.56, 13.20, and 13.10 sec, respectively. After 3 hr, the ethyl acetate fraction of leaves attained a much higher analgesic effect compared with that of acetylsalicylic acid (26.63 and 25.03 sec, respectively), followed by total stem extract, polysaccharides, petroleum ether fraction of leaves, total leaf extract, and aqueous fraction of leaves (21.50, 17.90, 17.03, 16.53, and 15.33 sec, respectively).

After 5 hr, the ethyl acetate fraction of leaves and total stem extract showed the highest analgesic activity among all extracts and fractions of *P. americana* (28.63 and 28.55 sec, respectively), with percentage of protection against external stimulus 137.95 and 137.12 %, respectively, which is higher than acetylsalicylic acid activity (27.56 sec and 129.1%). In addition, polysaccharides, total leaf extract, petroleum ether, and aqueous fractions of leaves showed marked analgesic effects (18.66, 17.90, 17.83, and 17.63 sec, respectively).

***Antipyretic activity***

Different extracts and fractions of leaves and stems of *P. americana *showed a marked and significant antipyretic activity by reduction of yeast-induced pyrexia (Table 8).

After 0.5 hr, no tested extracts and fractions showed significant decrease in the temperature compared with acetylsalicylic acid.

After 1 hr, the crude polysaccharides of the stem exhibited significant antipyretic activity (38.67 °C). In the same way, petroleum ether and ethyl acetate fractions of leaves, in addition to the total stem extract showed similar antipyretic effect (38.83 °C). Additionally, the aqueous fraction of leaves and total leaf extract showed no effect in the same time interval (39.16 and 39.32 °C, respectively).

Both total stem extract and crude polysaccharides attained the normal temperature after 3 hr and maintained their antipyretic activity up to 5 hr from the beginning of the experiment.

It is noteworthy that after 5 hr, all tested extracts and fractions attained the normal levels of rectal temperature (36.5-37 °C) except for the total leaf extract that showed very weak antipyretic activity where the rectal temperature decreased without arriving at the normal level even after 5 hr from its administration.

***Antihyperglycemic activity***

The different extracts and fractions of leaves and stems of *P. americana* exhibited antihyperglycemic activity by inhibition of alloxan-induced diabetes (Tables 9 and 10).

The total stem extract showed significant antihyperglycemic activity with a significant reduction in the level of blood glucose by 37.93 %, with rapid onset after 0.5 hr. Its effect was improved progressively until arriving at the highest level after 4 hr (59.05%) and then maintained for the remainder of the test. The total leaf extract was the most potent, displaying a significant decrease in the level of blood glucose especially after 3 hr (62.93%), which increased progressively until arriving at the highest level (76.67%) after 4 hr and then maintained for the rest of the test. The petroleum ether, ethyl acetate, and aqueous fractions of leaves, and crude polysaccharide of the stem also showed a moderate gradual decrease in the level of blood glucose after 3 hr with a percentage of reduction of 36.10, 34.15, 28.78, and 53.17, respectively. Their effects were increased progressively till the highest level reached after 4 hr and then maintained for the rest of the experiment. 

## Discussion

The anti-inflammatory potential of *P. americana* in rats using the paw edema test induced by carrageenan (acute inflammatory model) was investigated. The results showed the promising anti-inflammatory action of ethyl acetate fraction and total stem extract. The paw edema test induced by carrageenan is widely recognized as a sensitive phlogistic method to investigate the powerful anti-inflammatory drugs, mainly the nonsteroidal one (22). The edema induced by injection of carrageenan in rats is mediated by the release of 5-hydroxytryptamine and histamine during the first hour, and then the increased vascular permeability is maintained by the release of kinin until 2.5 hr. The mediator appears to be a prostaglandin released from 2.5 to 6 hr, which is closely related to leucocyte migration into the inflamed location (23). The anti-inflammatory efficacy of extracts of *P. americana* is due to the presence of phytochemical metabolites in these extracts, including phenolics, tannins, and flavonoids, which are documented to possess potent anti-inflammatory properties by inhibiting pathways of prostaglandin (24, 25). Additionally, its effect is due to the presence of fatty acids which were identified by GC-MS analysis of its saponifiable matter, that exhibited anti-inflammatory activity causing a reduction of systemic inflammation through significant inhibition of the inflammatory markers (26).

The analgesic effect of drugs can be evaluated by several models. The stimulus can be mechanical (tail or paw pressure tests), thermal (tail flick, tail immersion, and hot plate tests), electrical (stimulation of tail, paw, or dental pulp), or chemical (formalin and writhing tests) (27). The hot plate test is an appropriate method for the assessment of centrally-acting analgesics. It appears that the nociceptors are sensitized by sensory nerves. In this model, the contribution of endogenous substances like prostaglandins is reduced (28). The ethyl acetate fraction of leaves and total stem extract of *P. americana* exhibited the highest analgesic activity with percentage of protection against external stimulus at 137.95 and 137.12 %, respectively, in centrally active analgesic methods.

Fever could be caused by infection or one of the sequels of damage to tissue, rejection of graft, and/or other states of disease. Antipyretics are substances that decrease the elevated temperature of the body. Pyrexia induced by yeast is referred to as pathogenic fever and its etiology requires prostaglandin production, which set the thermoregulatory center at a lower temperature. Prostaglandins, which are mainly the most potent pyretic agent, tend to be a final pathway responsible for development of fever caused by several pyrogens. In general, antipyretic activity is one of the properties of non-steroidal anti-inflammatory drugs, due to their inhibitory effect on prostaglandin biosynthesis in the central nervous system (29). Both the total stem extract and crude polysaccharides possessed a significant antipyretic potential, in elevation of body temperature (39.8 °C) induced by yeast in rats, as the temperature was decreased to 37.0 °C, and the effects are comparable to the reference antipyretic drug (acetylsalicylic acid), which reduced the temperature to 36.4 °C. It appears that the observed antipyretic activity of *P. americana* may be due to prostaglandin synthesis inhibition. The antipyretic potential of extracts that contain flavonoids and saponins has been reported in various studies (30–33). Therefore, the activity may be due to presence of the above group of phytoconstituents in *P. americana*. 

Alloxan induces diabetes by killing the pancreatic insulin-secreting cells, resulting in hypoinsulinemia and hyperglycemia (34). Alloxan triggers hyperglycemia by means of specific cytotoxic effect on pancreatic beta cells. Its cytotoxicity is due to *in vivo* and *in* *vitro* production of free radicals (35). Finally, the potent antihyperglycemic activity of the different extracts and fractions of *P. americana* leaves could be owing to their high flavonoid content, especially isorhamnetin, luteolin, rutin, quercetin, and apigenin (16). Flavonoids are a type of phenolic secondary metabolite found in plants that are extensively spread in nature and possess a potent hypoglycemic potential, as shown in many models of experiments (36, 37). The hypoglycemic activity of flavonoids is owing to their ability to inhibit the absorption of glucose or to increase the tolerance of glucose. Furthermore, flavonoids may act as insulin mimetics, stimulate peripheral tissues’ uptake of glucose and regulate the expression and/or activity of the rate-limiting enzymes engaged within the pathway of carbohydrate metabolism (37). On the other hand, the potent effect of the crude polysaccharides might be attributed to the decreased absorption of carbohydrates from the gut.

**Table 1:** A list of fatty acids identified as methyl esters from the saponifiable matter of *Persea americana* leaves by GC/MS

**Relative Area %**	**R**_t_ ** (min)**	**Mol.** **weight**	**Mol. formula**	**Compounds**	**Peak** **no.**
0.59	10.419	214	C_13_H_26_O_2_	Dodecanoic acid methyl ester	**1**
4.30	16.127	242	C_15_H_30_O_2_	Tetradecanoic acid methyl ester	**2**
0.43	21.140	268	C_17_H_32_O_2_	9-Hexadecenoic acid methyl ester	**3**
0.83	21.426	282	C_19_H_38_O	2-Heptadecyloxirane	**4**
55.07	21.524	270	C_17_H_34_O_2_	Hexadecanoic acid methyl ester	**5**
1.86	22.933	284	C_18_H_36_O_2_	Heptadecanoic acid methyl ester	**6**
9.92	24.063	294	C_19_H_42_O_2_	9,12-Octadecadienoic acid methyl ester	**7**
14.89	24.170	348	C_23_H_40_O_2_	8,11,14-Docosatrienoic acid methyl ester	**8**
1.74	24.244	282	C_18_H_34_O_2_	9-Octadecenoic acid methyl ester	**9**
4.89	24.594	298	C_19_H_38_O_2_	Octadecanoic acid methyl ester	**10**
0.72	28.480	346	C_23_H_38_O_2_	6,9,12,15- Docosatetraenoic acid methyl ester	**11**
0.49	28.681	292	C_19_H_32_O_2_	9,12,15-Octadecatrienoic acid methyl ester	**12**
0.89	29.202	326	C_21_H_42_O_2_	Eicosanoic acid methyl ester	**13**
3.39	30.079	254	C_16_H_30_O_2_	4,8,12-Trimethyltridecan-4-olide	**14**
67.6%	Total identified long-chain saturated fatty acids				
32.4%	Total identified long-chain unsaturated fatty acids				

**Table 2:** A list of identified components of ethyl acetate fraction (as silylated derivatives) of *Persea americana* leaves by GC/MS analysis

**Characteristic peaks (%)**	**Base peak**	**Rt (min)**	**Mol. formula**	**M.W**	**Compounds**	**No.**
179(89.8%), 77(80.2%), 135(50.3%), 51(25.6%), 45(15.5%)	105	7.88	C_10_H_14_O_2_Si	194	Benzoic acid trimethylsilyl ester	**1**
174(33.7%), 59(23.6%), 75(22.4%), 147(18.2%), 86(13.2%)	73	8.03	C_13_H_33_NO_2_Si_3_	319	Butanoic acid, 4-[bis(trimethyl silyl)amino]-, trimethylsilyl ester	**2**
147(49.5%), 205(38.4%), 103(29.6%), 117(28.7%), 45(20.6)	73	8.67	C_12_H_32_O_3_Si_3_	308	Trimethylsilyl ether of glycerol	**3**
129(38.8%), 147(22.5%), 103(17.9%), 75(14.2%), 205(11.1%)	73	8.85	C_15_H_34_O_5_Si_3_	378	D-Arabino-Hexonic acid, 3-deoxy-2,5,6-tris-O-(trimethylsilyl)-, γ-lactone	**4**
73(72.7%), 75(26.7%), 148(16.4%), 247(15.2%), 45(14.6%)	147	8.88	C_10_H_22_O_4_Si_2_	262	Butanedioic acid, bis(trimethyl silyl) ester	**5**
41(92.7%), 79(88.5%), 93(66.1%), 91(57.3%), 95(42%)	43	12.98	C_15_H_24_O	220	Caryophyllene oxide	**6**
73(99.7%), 129 (70%), 204(44.2%), 218(37.9%), 132(32.7%)	217	18.81	C19H46O6Si4	482	1.Methyl 2,3,5,6-tetrakis-O-(trimethylsilyl)hexofuranoside	**7**
204(72.4%), 217(37.6%), 147(24.7%), 191(22%), 205(16.6%)	73	19.38	C17H42O5Si4	438	D-Xylopyranose, 1,2,3,4-tetrakis-O-(trimethylsilyl)-	**8**
204(48.7%), 191(25.1%), 217(24.7%), 147(16.6%), 75(11.2%)	73	20.27	C17H42O5Si4	438	Lyxose, tetra-(trimethylsilyl)-ether	**9**
73(25%), 75(25%), 144(18%), 123(17%), 43(89%)	143	23.03	C23H48OSi	368	Phytol, trimethylsilyl ether	**10**
231(64%), 147(29%), 205(26.7%), 129(17.9%), 232(13%)	73	26.19	C17H44O4Si4	424	Pentitol, 3-desoxy-tetrakis-O-(trimethylsilyl)-	**11**
313(70%), 147(60%), 97(40.9%), 57(38.1%), 83(38.1%)	73	29.18	C23H52O2Si2	416	n-Heptadecan-1,2-diol,bis-(trimethylsilyl)ether	**12**
73(93.5%), 147(88.9%), 81(51.8%), 95(35.5%), 41(25.5%)	75	29.29	C15H32OSi2	284	Borneol, pentamethyldisilanyl ether	**13**

**Table 3:** A list of identified components of aqueous fraction (as silylated derivatives) of *Persea americana* leaves by GC/MS analysis

**Characteristic peaks (%)**	**Base peak**	**R**_t_ ** (min)**	**Mol. formula**	**M.W**	**Compounds**	**No.**
31(87.6%), 126 (81.2%), 29(73.1%), 127(70%), 43(61.41%)	96	3.64	C_8_H_12_F_3_NO_6_	275	N-Trifluoroacetyl-D-glucosamine	**1**
73(72.7%), 75(26.7%), 148(16.4%), 24.7(15.2%), 45(14.6%)	147	8.88	C_10_H_22_O_4_Si_2_	262	Succinic acid, di(trimethyl silyl) ester	**2**
147(34.7%), 205(22%), 103(21.4%), 117(20.9%), 45(12.7%)	73	18.13	C_12_H_32_O_3_Si_3_	308	Trimethylsilyl ether of glycerol	**3**
193(97.6%), 147(26.2%), 75(17.7%), 194(17%), 45(13.8%)	73	18.74	C_15_H_28_O_2_Si_2_	296	2-Phenyl-1,2-bis(trimethylsilyloxy)propane	**4**
174(29%), 75(16.5%), 74(8.1%), 204(6.2%), 45(5.1%)	73	21.16	C_13_H_28_O_4_Si_2_	304	Malonic acid, bis(2-trimethylsilylethyl ester	**5**
103(26.4%), 147(14.5%), 217(9.8%), 45(8.2%), 74 (7.8%)	73	24.83	C_20_H_52_O_5_Si_5_	512	Ribitol, 1,2,3,4,5-pentakis-O-trimethylsilyl))	**6**

**Table 4:** Anti-inflammatory activity of different extracts and fractions of the leaves and stems of *Persea americana* using carrageenan-induced paw edema method

**Thickness of the paw (mm) **								**Dose mg/kg**	**Group**
**5 h**	**4 hr**	**3 hr**	**2 hr**	**1 hr**	**0.5 hr**	**Zero time**		
6.90±0.10		6.78±0.12	7.02±0.15	7.40±0.10	7.52±0.10	7.61±0.15	7.55±0.15	-	**Control**
3.94±0.10		4.76±0.11	5.54±0.11	5.98±0.05	6.53±0.10	7.16±0.15	7.19±.10	8	**Indomethacin**
5.54±0.11***		5.74±0.15**	6.23±0.11**	6.5±0.05**	7.1±0.11	7.5±0.05	7.41±0.05	100	**Total leaf extract**
4.24±0.15***		4.55±0.11***	5.85±0.10**	6.51±0.15**	6.82±0.11*	7.12±0.15	7.18±0.11	100	**Pet. ether fraction of leaves**
3.52±0.10***		4.35±0.15***	5.21±0.11***	6.13±0.05***	6.41±0.15**	6.82±0.11*	7.39±0.11	100	**Ethyl acetate fraction of leaves**
5.0±0.15***		5.52±0.11**	6.0±0.11**	6.73±0.11*	6.70±0.11**	7.21±0.10	6.96±0.15	100	**Aqueous fraction of leaves**
3.62±0.11***		3.81±0.05***	4.11±0.15***	5.82±0.05***	6.51±0.05***	7.01±0.15*	6.54±0.15	100	**Total stem extract**
4.81±0.05***		5.0±0.11***	5.54±0.11**	6.02±0.10***	6.53±0.11**	6.70±0.11**	6.92±0.05	100	**Crude polysaccharides of the stem**

The results were presented as the mean±SE. (standard error) where n=5. Differences with respect to the control group were evaluated using student's T-test (*P<0.05, **P<0.01, and ***P<0.001)

**Table 5.** Percentages of edema inhibition of different extracts and fractions of leaves and stems of *Persea americana*

**Percentages of inhibition (%)**							**Dose mg/kg**	**Group**
**5 hr**	**4 hr**	**3 hr**	**2 hr**	**1 hr**	**0.5 hr**	**Zero time**		
42.90	29.79	21.08	19.19	13.16	5.91	4.77	8	**Indomethacin**
19.71	15.34	11.25	12.16	5.59	1.45	1.85	100	**Total leaf extract**
38.55	32.89	16.67	12.03	9.31	6.44	4.90	100	**Pet. ether fraction of leaves**
48.99	35.84	25.78	17.16	14.76	10.38	2.12	100	**Ethyl acetate fraction of leaves**
27.54	18.58	14.53	9.05	10.90	5.26	7.81	100	**Aqueous fraction of leaves**
47.54	43.81	41.45	21.35	13.43	7.88	13.38	100	**Total stem extract**
30.29	26.25	21.08	18.65	13.16	11.96	8.34	100	**Crude polysaccharides of the stem**

**Table 6:** Analgesic activity of different extracts and fractions of leaves and stems of *Persea americana* using the hot plate method

Rectal temperature (°C)									Dose mg/kg	Group
**5 h**		**4 hr**	**3 hr**	**2 hr**	**1 hr**	**0.5 hr**		Pre-drug	Before yeast injection	
39.93±0.25	39.93±0.18	39.82±0.17	39.79±0.70	39.52±0.22		39.34±0.54	39.17±0.34	36.92±0.23	-	Control
35.41±32***	35.52±0.24***	36.40±0.19*	37.50±0.0.15**	37.88±0.28		38.40±0.12	38.86±0.16	36.53±0.13	100	Acetylsalicylic acid
38.50±0.32***	38.74±0.22*	38.83±0.25**	39.17±0.25*	39.32±0.13*		39.50±0.10	38.86±0.28	36.33±0.39	100	Total leaf extract
36.50±0.15**	37.33±0.19**	37.83±0.41**	38.5±0.29**	38.83±0.15*		39.16±0.51	38.88±0.37	36.82±0.36	100	Petroleum ether fraction of leaves
37.0±0.22***	37.50±0.12**	37.78±0.23**	38.5±0.40***	38.83±0.24***		39.0±0.15	38.92±0.13	36.75±0.28	100	Ethyl acetate fraction of leaves
36.67±0.36**	37.21±0.13***	38.0±0.13*	38.52±0.36**	39.16±0.23*		39.23±0.38	39.02±0.29	36.75±0.40	100	Aqueous fraction of leaves
36.50±0.44***	36.83±0.15**	37.0±0.33***	37.50±0.29*	38.83±0.44**		39.17±0.42	38.60±0.47	36.21±0.25	100	Total stem extract
36.50±0.33***	36.83±0.44***	37.0±0.51***	37.32±0.24*	38.67±0.33***		39.23±0.14	38.92±0.49	36.83±0.15	100	Crude polysaccharides of the stem

All experimental groups were composed of 5 animals. The results were presented as the mean±SE. (standard error). Differences with respect to the control group were evaluated using one-way ANOVA followed by Dunnett's test (*P<0.05, **P<0.01, and ***P<0.001)

**Table 7:** Percentage of protection against external stimulus of different extracts and fractions of the leaves and stems of *Persea americana*

Reaction time in seconds							Dose mg/kg	Group
5 h	4 **hr**	3 **hr**	2 **hr**	1 **hr**	0.5 **hr**	Zero time		
12.03±17.32	11.70±4.04	11.30±7.75	11.00±7.76	10.80±10.15	10.56±8.35	10.40±9.29	-	Control
27.56±15.28	25.43±27.96	25.03±21.79	23.53±28.37	17.16±23.31	17.00±24.64	12.06±17.61	100	Acetylsalicylic acid
17.90±5.292*	17.30±4.93***	16.53±8.19**	15.86±2.03**	13.56±17.94	12.86±16.5	10.23±9.95	100	Total leaf extract
17.83±1.76***	17.46±3.18***	17.03±6.36**	15.30±7.02*	13.10±20.31**	12.30±20.23	10.20±9.16	100	Petroleum ether fraction of leaves
28.63±5.93*	28.30±6.43***	26.63±3.7***	23.36±18.81**	17.63±4.67**	11.00±11	10.40±10.4	100	Ethyl acetate fraction of leaves
17.63±8.11*	16.26±4.63**	15.33±4.67*	14.26±5.24*	13.20±5.51	12.16±15.63	11.90±4.16	100	Aqueous fraction of leaves
28.53±5.24***	26.00±10.82***	21.50±20.07**	16.83±15.38*	14.10±12.22	11.70±9.45	10.80±11.72	100	Total stem extract
18.66±5.55*	18.36±4.67***	17.90±5.77**	16.76±7.84**	15.73±15.19	13.26±22.32	12.06±15.3	100	Crude polysaccharides of the stem

All experimental groups were composed of 5 animals. The results were presented as the mean

**Table 8:** Antipyretic activity of different extracts and fractions of leaves and stems of *Persea americana *using yeast-induced pyrexia method

Percentage of protection against external stimulus (%)						Dose mg/kg		Group
5 h	4 **hr**	3 **hr**	2 **hr**	1 **hr**	0.5 **hr**	Zero time		
129.10	117.38	121.53	113.94	58.95	60.88	15.96	100	Acetylsalicylic acid
48.76	47.86	46.31	44.25	25.62	21.77	-1.61	100	Total leaf extract
48.20	49.29	50.73	39.09	21.30	16.40	-1.92	100	Petroleum ether fraction of leaves
137.95	141.88	135.69	112.43	63.27	4.10	0	100	Ethyl acetate fraction of leaves
46.54	39.03	35.69	29.7	22.22	15.14	14.42	100	Aqueous fraction of leaves
137.12	122.22	90.27	53.03	30.56	10.72	3.85	100	Total stem extract
55.13	56.98	58.41	52.43	45.68	25.55	15.96	100	Crude polysaccharides of the stem

All experimental groups were composed of 5 animals. The results were presented as the mean±SE. (standard error). Differences with respect to the control group were evaluated using student's T-test (*P<0.05, **P<0.01, and ***P<0.001)

**Table 9:** Antihyperglycemic activity of different extracts and fractions of leaves and stems of *Persea americana* using the alloxan-induced hyperglycemia method

Blood glucose level (mg/dl)							Dose mg/kg	Group
5 h	4 **hr**	3 **hr**	2 **hr**	1 **hr**	0.5 **hr**	Zero time		
199±7.9	200±3.5	203±5.7	203±5.9	205±5.5	210±5	210±2	-	Control
180±4.5	187±3.2	202±1.9	217±6.9	221±5	241±6.7	232±5.7	0.5	Glibenclamide
42±5.4**	49±0.9**	76±0.7**	123±45.5***	182±23.2*	236±32.4	242±21.7	100	Total leaf extract
99±18.5**	107±37.4*	131±22.1***	185±10.2**	197±17.9**	200±15.4	236±6.5	100	Petroleum ether fraction of leaves
107±15.4**	111±22.5**	135±11.3*	171±5.4*	199±9.1*	202±16.8	207±4.5	100	Ethyl acetate fraction of leaves
94±12.5***	100±14.5***	146±5.7***	160±18.5***	166±15.4***	212±4.7	231±15.2	100	Aqueous fraction of leaves
80±4.5**	86±11.5*	88±0.4*	111±17.6**	126±22.5**	133±54.6***	174±45	100	Total stem extract
91±5.9***	95±17.4***	96±10.5*	121±15.4***	155±33*	193±26.3**	195±15	100	Crude polysaccharide of the stem

All experimental groups were composed of 5 animals. The results are presented as the mean±SE. (standard error). Differences with respect to the control group were evaluated using one-way ANOVA followed by Dunnett's test (*P<0.05, **P<0.01, and ***P<0.001)

**Table 10:** Effect of different extracts and fractions of leaves and stems of *Persea americana* on lowering the blood glucose level percentage in hyperglycemic rats

lowering blood glucose percentage							Dose mg/kg	Group
5 hr	4 hr	3 hr	2 hr	1 hr	0.5 hr	Zero time		
10.95	10.95	1.46	-6.90	-8.87	-20.5	-16.59	0.5	Glibenclamide
76.67	76.67	62.93	39.41	10.34	-18	-21.61	100	Total leaf extract
49.05	49.05	36.10	8.87	2.96	0	-18.59	100	Petroleum ether fraction of leaves
47.14	47.14	34.15	15.76	1.97	-1	-4.02	100	Ethyl acetate fraction of leaves
52.38	52.38	28.78	21.18	18.23	-6	-16.08	100	Aqueous fraction of leaves
59.05	59.05	57.07	45.32	37.93	33.5	12.56	100	Total stem extract
54.76	54.76	53.17	40.39	23.65	3.5	2.01	100	Crude polysaccharide of the stem

## Conclusion

The petroleum ether, ethyl acetate, and aqueous fractions of *P. americana* leaves have been analyzed by the GC-MS method. Furthermore, the extracts, different fractions, and crude polysaccharides of the stem were studied for their analgesic, anti-inflammatory, antipyretic and antihyperglycemic efficiencies. These findings proposed that *P. americana* possessed analgesic, anti-inflammatory, antipyretic, and antihyperglycemic properties, which supported the traditional application of plant under study in treatment of pain, inflammation, fever, and diabetes. 
